# The Influence of Guanxi Between Boundary Spanners on Opportunistic Behaviors Based on the Theory of Reasoned Action Model

**DOI:** 10.3389/fpsyg.2022.851780

**Published:** 2022-03-03

**Authors:** Shu-kuan Zhao, Jia-ming Cai

**Affiliations:** School of Business and Management, Jilin University, Changchun, China

**Keywords:** Guanxi, boundary spanner, opportunistic behavior, collaborative innovation, theory of reasoned action

## Abstract

This study constructs a mechanism of the influence of Guanxi between boundary spanners on opportunistic behaviors in collaborative innovation projects based on the theory of reasoned action model. The study conducts a survey in the automobile industry in Changchun, Jilin Province, China, and analyzes the research data using the structural equation model. The findings show that Guanxi has a negative an significant influence on opportunistic behavior attitudes and subjective norms., Guanxi has the greater influence on subjective norms than attitudes. Then, opportunistic behavior attitudes and subjective norms positively influence intentions. The influence of subjective norms is stronger. The attitudes and subjective norms of opportunistic behaviors also play mediation roles. Furthermore, opportunistic behavior intentions have a positive and significant influence on behaviors. In short, the study’s findings reveal a mechanism of Guanxi between boundary spanners influencing opportunistic behaviors of boundary spanners. It also provides a reference for corporate managers to govern opportunistic behaviors of collaborator while inhabiting opportunistic behaviors of their own boundary spanners.

## Introduction

Innovation is a path for companies to gain excessive profits (Vrontis and Christofi, 2021). According to resource dependency theory, few organizations can be completely self-sufficient. Organizations must exchange resources with outside world to survive and thrive (Pfeffer and Salancik, 1979). As product iteration speed increased and market competition intensifies, internal resources cannot support enterprises in pursuing long-term innovation. As a result, many enterprises seek partners with complementary resources and capabilities to carry out collaborative innovation (Solaimani and van der Veen, 2021), sharing costs, risks, and resources and improve innovation success rates (Bogers et al., 2018).

After >50 years of development, China’s automobile industry has achieved a competitive advantage globally. Despite being hit by COVID-19, car sales in China reached 25.311 million (C.A.O.A. Manufacturers, 2021). For many years, it has been ranked first in the world (C.A.O.A. Manufacturers, 2021). Despite the fact that the domestic auto market is not yet saturated, the Chinese auto industry continues to face challenges. For starters, traditional fuel vehicle manufacturers face production challenges because new energy vehicles are the future development direction (C.A.O.A. Manufacturers, 2021). Second, the global supply chain has been severely affected by COVID-19. The shortage of automobile chip supply and continued high prices of raw materials have raised the cost pressure on enterprises (C.A.O.A. Manufacturers, 2021). Under such intense competition, collaborative innovation in the automobile industry is required, such as vertical and horizontal supplier integration by vehicle manufacturers. Collaborative innovation improves automakers` ability to deal with market challenges and boosts competitiveness (Ding et al., 2019). China FAW Group Co. Ltd. (abbreviated “FAW”) in Changchun, for example, is a large-scale vehicle manufacturer in China. It widely employs the strategy of collaborative innovation. For example, the R&D team sent by FAW’s steel suppliers Baosteel Group Co., Ltd. and Angang Co., Ltd. intervenes in the primary stage of FAW’s product design, which can shorten new product development time and reduce the risk (A.I. Research, 2015). However, opportunism is frequently caused by the uncertainty of innovation, information asymmetry, and specific asset investment, which limits the sustainability and output of collaboration (Tangpong et al., 2015; Villena et al., 2021).

Because opportunism in inter-firm relationships is commonly seen as an obstacle to fostering collaboration, researchers have paid increased attention to curtailing opportunism in such conditions. Early studies, based on the theory of transaction cost economics, considered economic forces, such as contractual safeguarding, shared ownership, asset-specific investments, and managerial governance. All of them are based on the formal construct (Luo et al., 2015). However, recent research has gradually revealed that forces based on transaction cost theory are woefully inadequate for long-term oriented collaborative innovation. Economic cooperation is gradually becoming ingrained in social relationships (Granovetter, 1985). As a result, informal governance forces based on social exchange theory, such as Guanxi, trust, and justice, can serve as a viable alternative to formal constructs. The Chinese-specific concept Guanxi is thought to perhaps serve as an informal governance force of opportunistic behaviors in Chinese inter-organization relationships. Some scholars have found that inter-organizational Guanxi plays a vital role in building inter-organizational trust, and governing the opportunism of collaborators in the Chinese context (Su et al., 2008; Xue et al., 2017; Yang et al., 2021; Zhang et al., 2021b). There are some major gaps in existing research on inter-firm opportunism and Guanxi. To begin, existing research focuses on Guanxi as a governance force at the inter organizational level while ignoring the individual level. Second, boundary spanners are front-line employees who interact with their counterparts on behalf of the firms (Mäkelä et al., 2019). They can sometimes be opportunistic in collaboration projects for the benefit of their own firms. The opportunistic behaviors of boundary spanners can have a strong negative effect on the longevity of collaborative innovation projects. However, existing study do not notice the boundary spanners` opportunistic behaviors.

Therefore, this study will examine the influence of Guanxi between boundary spanners on the opportunistic behaviors in automobile industry collaborative innovation projects in Changchun, Jilin Province, China. The research will present the theory of reasoned action (TRA) model to build the mechanism of Guanxi and opportunistic behaviors. Following a review of the literature, the researchers developed a research model and proposed some hypotheses about “Guanxi,” “opportunistic behavior attitudes,” “opportunistic behavior subjective norms,” “opportunistic behavior intentions,” and “opportunistic behaviors.” The mechanism of Guanxi influencing opportunistic behaviors of boundary spanners will be revealed by TRA’s construction of the research model. Through data collection and analysis, this study will further confirm whether Guanxi influences opportunistic behavior intentions through opportunistic behavior attitudes and subjective norms, and identify which one plays a pivotal mediation role. It will also look into whether opportunistic behavior intentions can predict actual behavior. As a result, this study provides managers with a new perspective on understanding opportunistic behaviors in collaborative innovation projects. It also serves as a reminder to managers to pay closer attention to the role of boundary spanners. They should encourage boundary spanners to cultivate Guanxi appropriately.

## Literature Review and Hypotheses Development

### China Changchun Automobile Industry Collaborative Innovation Background

Changchun, the capital of Jilin Province in northeastern China, is recognized as the Automobile City of China. The automobile industry is the leading industry in Changchun and Jilin Province (C.C.B.O. Statistics, 2021; J.P.B.O. Statistics, 2021). According to statistics, the automobile industry accounts for 69.9% of the city’s industry (C.C.B.O. Statistics, 2020). FAW, headquartered in Changchun, is a vehicle manufacturer ranked 66th on the Fortune 500 list (FAW, 2021). The company’s sales volume reached 3.706 million vehicles in 2020. The company’s operating revenue is 697.42 billion yuan (FAW, 2021). At present, FAW’s business includes three independent brands, namely, Hongqi, Jiefang, and Pentium, as well as two joint venture brands, namely, FAW-Volkswagen and FAW-Toyota. Brand values of Hongqi and Jiefang ranked first among domestic independent passenger cars and commercial vehicles (FAW, 2021). According to C.A.O.A. Manufacturers (2021) statistics, FAW-Volkswagen sold 2.07 million vehicles in 2020, ranking first among domestic automobile manufacturers.

According to the introduction of the China Changchun Automotive Economic and Technological Development Zone (2017), FAW has driven the extension of the automobile industry chain in Changchun. In the Changchun Auto Economic and Technological Development Zone, there are currently more than 300 automobile components suppliers. Many influential automobile component firms, such as FAW Fuwei, Jack-Sell Air Conditioning, FAW Casting, FAW Forging, and FAW Abrasive Center, form a complete supporting system and a cluster of components manufacturing enterprises with competitive advantages in China. Furthermore, the FAW Technical Centre in Changchun, China Machinery Industry’s Ninth Design Institute, and Jilin University’s School of Automobile Engineering constitute China’s most densely populated area for automobile R&D and educational institutions.

The automobile industry is a technology-intensive industry that should pay great attention to innovation. Collaborative innovation can achieve the coordinated development of vehicle manufacturers and spare parts manufacturers. It also accelerates product iteration and industrial upgrading (Grather et al., 2015). The entire automobile industry chain, as well as intensive automobile R&D educational institutions, can provide an excellent foundation for collaborative innovation (De Wit-de Vries et al., 2019). The Changchun automobile industry has undertaken many collaborative innovation projects due to its unique industrial and scientific resources. Many vertical and horizontal collaborative innovation projects have been conducted between the upstream and downstream of the industry chain and between the automobile manufacturers and R&D institutions. However, some factors, such as information asymmetry and specific asset investment, can occasionally lead to opportunism in collaborative innovation projects. Opportunism impedes the long-term development of collaborative innovation relationships and has an effect on the quality of innovation outcomes. It will dampen the enthusiasm of enterprises to carry out collaborative innovation in the long run (Oliveira and Lumineau, 2018).

### Guanxi Between Boundary Spanners

Guanxi, rooted in traditional Chinese Confucian philosophy, shapes Chinese people’s thinking patterns and behaviors (Su et al., 2008). Guanxi is a primary mode of social bonding and human interaction. It is a network of human connections that allows people to benefit from being connected to others or organizations (Park and Luo, 2001). Guanxi has three main operational features. To begin with, reciprocity is the fundamental principle (Zhang et al., 2021b). Guanxi is under an intangible obligation to repay the favor (Hwang, 1987). Otherwise, Guanxi will be affected if one party does not return the favor and assist the other in their time of need. Second, Guanxi is a long-term mindset. Maintaining harmonious and stable relationships is an essential guideline for Chinese exchange (Su et al., 2008). Finally, Guanxi is an expression of emotion but also utilitarian considerations. The need for “favors” is particularly evident in business (Park and Luo, 2001).

In the commercial activities of Chinese enterprises, the development of Guanxi between boundary spanners has become a common phenomenon (Cai Shaohan and Zhilin, 2016). Qian et al. (2016) believed that Guanxi follows the principle of reciprocity. To gain the support of collaborative enterprises, boundary spanners take the initiative to care for and help each other in daily interaction. They will consciously restrain their behaviors from hurting the collaborative enterprises to avoid losing more long-term benefits. Second, Shen et al. (2019) argued that the Guanxi of boundary spanners increases the long-term orientation of the collaboration. During the collaboration process, it allows them to gain a better understanding of one another. It can improve their predictability and contribute to the development of reasonable levels of trust and commitment. Furthermore, Su et al. (2008) demonstrated that Guanxi between boundary spanners promotes high-quality communication and collaboration between the two parties. They actively take on their responsibilities and are willing to work together to solve problems, reducing the possibility of conflict and establishing a harmonious collaborative relationship. In this study, Guanxi is defined as the intimacy and permanence of the private relationship interaction between boundary spanners of both parties outside of their work in the collaborative innovation project of the automobile industry chain.

### Opportunistic Behaviors

Williamson (1985) defines opportunism as “subterfuge for personal gain” or “incomplete or distorted disclosure of information, especially where it is intentionally misleading, distorted, disguised, confused or otherwise obfuscated.” Opportunistic behavior is broadly defined in the context of business cooperation, as “deceptive behavior by a cooperating firm in pursuit of its interests at the expense of other cooperating firms” (Das and Rahman, 2009). Non-compliance with implicit or explicit contractual norms of cooperation, abuse of power, concealment or distortion of information, non-performance of commitments made, non-undertaking of cooperation obligations, and improper appropriation of cooperation achievements are examples of opportunistic behaviors (Luo, 2007a). In this study, opportunistic behaviors refer to deceptive actions by boundary spanners in collaborative innovation projects to harm the other company within the range of their authority to safeguard the interests of their own company, such as neglecting to share knowledge resources and hiding resources needed by the partner.

Concerning the study of opportunism, most existing research has examined how to govern the opportunism of collaboration partners, mainly under economic exchange and social exchange logic (Luo et al., 2015). Luo (2007b) summarized existing studies and concluded four “ordering” systems (1) contractual ordering, (2) structural ordering, (3) relational ordering, and (4) justice ordering. The first two represent economic exchange logic. The logic of social exchange is represented by the latter two. Furthermore, some scholars, such as Xue et al. (2018), argued that trust is also a governance mechanism under the social exchange logic. Then, Brito and Miguel (2017) proposed that inter-firm dependencies can also be considered a type of governance. Scholars have extensively researched these governance models. First, Yu et al. (2020) explored the effect of contractual governance on the opportunism of partners in cross-cultural contexts of industry chain cooperation. Second, Wang et al. (2016) discussed the role of institutional forces in the governance of opportunism of local suppliers. Furthermore, Zhang et al. (2021b) investigated the efficacy of relationship governance in various institutional settings. Most existing studies have looked at how to reduce opportunism among collaborators from a governance standpoint, and the mechanisms that cause opportunism are insufficient. This study will discuss the mechanism of opportunistic behaviors. It will explore how the Guanxi between boundary spanners affects their opportunistic behaviors.

### Theory of Reasoned Action

The TRA proposed by Ajzen and Fishbein (1977) is considered one of the most fundamental and influential theories in cognitive behavior research. The theory is used to investigate how people change their behaviors by changing their intentions. Human behavior, according to this belief, is the result of rational thought. Attitude, subjective norm, behavioral intention, and behavior are the variables that explain and predict individual behaviors. The perception and evaluation of the execution of a specific behavior are referred to as attitude. It also includes consideration of the subsequent outcome. The subjective norm is the opinion and perception of social pressure by the important people around when performing a specific behavior. People are more likely (or less likely) to engage in a behavior if important people around them support (or oppose) it. Furthermore, the intention is the desire to engage in a specific act, which is required for the act to be performed. Attitude and subjective norms influence the intention (Ajzen and Fishbein, 1977).

TRA has been used in many areas, including applications to explore some behaviors of employees in the workplace (Chen et al., 2021). For example, Ng (2020) applied the TRA to explain employees’ knowledge-sharing behaviors. The study also looked at the moderation effect of trust. Second, Khalil et al. (2021) used the extended TRA to investigate the influences of individual collectivism, uncertainty avoidance, attitudes, and subjective norms on knowledge-sharing intentions and behaviors. Furthermore, Hartanti et al. (2021) used TRA to explain the development of employee information security behaviors. Finally, Fu et al. (2020) combined TRA with information processing theory to investigate government caregivers’ innovation adoption behaviors. As a result, using TRA to discuss employee behaviors in the workplace is a good idea. Consequently, it is equally feasible for this study to apply TRA to explore the opportunistic behaviors of boundary spanners in collaborative innovation projects.

### Research Hypotheses

#### Guanxi and TRA Model Hypotheses

The social embeddedness theory holds that economic behaviors are embedded in personal social relations and ties (Granovetter, 1973, 1985). As a result, scholars have investigated the influence of Guanxi on employee behaviors in the workplace. First, the study of Wang et al. (2019) found that leader–subordinate Guanxi increases constructive behavior in subordinates through psychological empowerment. Second, according to Zhang and Deng (2016), leader–subordinate relationships reduce counterproductive behavior by increasing employee job satisfaction. Furthermore, He et al. (2019) discovered that forcing employees to adopt organizational citizenship behaviors can result in counterproductive behaviors—silent behaviors. When examining the effect of Guanxi on employees’ behaviors, most studies have focused on the Guanxi between leaders and subordinates. There is little research concentrated on the Guanxi between boundary spanners. There is also a lack of research concentrated on the relationship between Guanxi and opportunistic behaviors of boundary spanners. Taking TRA into account, it is possible to explain the mechanism of behavior generation. According to this study, it is also appropriate for explaining employees’ workplace behaviors. The TRA will be used in this study to investigate the influence mechanism between Guanxi and opportunistic behaviors.

In collaborative innovation projects, boundary spanners from two enterprises both represent their own company. So the interests of the individual and the company are bounded and same (Cai et al., 2021). The following analysis is made on the basis of this premise and three main characteristics of the Guanxi operation. First, if the boundary spanner engages in opportunistic behaviors to harm the collaborators, it will also hurt the boundary spanner from the collaborator enterprise. Opportunistic behavior is a violation of the reciprocity principle (Hwang, 1987). Second, the Chinese code of conduct emphasizes the importance of long-term and stable Guanxi (Su et al., 2008). Taking advantage of opportunities may benefit their businesses in the short term. Nonetheless, once discovered by the boundary spanner from the collaborator enterprise, it will harm Guanxi’s long-term development in the future. Finally, people show affection in the expectation that the other person will do them “favors” based on their business Guanxi (Park and Luo, 2001). In comparison, enterprises taking opportunistic behaviors will not get favors from collaborators because opportunistic behaviors hurt the other party.

To sum up, it can be inferred that the better the Guanxi between boundary spanners, the lower their attitudes toward opportunistic behaviors. Therefore, this study proposes the following hypothesis.

*H1:* Guanxi between boundary spanners will negatively and significantly affect their opportunistic behavior attitudes in a collaborative innovation project.

Xiaotong (1999), a famous sociologist and anthropologist, suggested that Chinese people’s pattern of dealing with others follows a “hierarchical order.” Individuals apply the Guanxi norms of reciprocity, long-term orientation, “retribution,” and “loyalty” to those close to them (Hwang, 1987). Individuals always disregard petty profits in the short term when guided by long-term orientation. As a result, both parties should treat each other with kindness and loyalty and avoid hurting each other behind each other’s backs (Luo et al., 2016). The Guanxi norm is widely accepted in society. The word-of-mouth system monitors the application of relationship norms (Lee and Lee, 2017). In other words, people around them will assess an individual’s adherence to Guanxi norms in social communication. As a result, the word-of-mouth shapes their reputation, which affects individuals’ long-term access to social resources (Chen and Wu, 2011). The boundary spanners’ opportunistic behaviors for the benefit of their own company are against the principle of long-term orientation and reciprocity in the personal Guanxi interaction. If they behave opportunistically, they will be under the pressure of public judgment. People around them will judge them as “faithless,” “petty,” and “short-sighted.” In the long run, their reputation will deteriorate, leading to exclusion from the “circle.” They can no longer use the “circle’s” network of connections, which will have bad effect on many aspects of life and work.

In summary, when boundary spanners have good Guanxi, they are subject to tremendous social pressure to behave opportunistically in collaboration. As a result, this study contends Guanxi between boundary spanners will have a negative influence on their subjective norms of opportunistic behavior. The following hypothesis is proposed in this study.

*H2:* Guanxi between boundary spanners will negatively influence their opportunistic behavior subjective norms.

#### Hypotheses Related to the TRA Model

TRA proposes that an individual’s attitudes and subjective norms toward a behavior influence the intention to adopt the behavior (Ajzen and Fishbein, 1977). For example, Ng (2020) showed that employees’ knowledge-sharing attitudes, subjective norms of knowledge sharing positively affect intentions. Furthermore, Siponen et al. (2014) discovered that employees’ attitudes and subjective norms toward information security policies positively influence behavioral intentions. Finally, one study looked into employees’ green IT usage habits (Mishra et al., 2014). According to the findings, usage attitudes and subjective norms positively predicted usage intention. In summary, opportunistic behavior intentions are higher when boundary spanners’ opportunistic behavior attitudes and subjective norms are higher in collaborative innovation projects. Therefore, this study proposes the following hypotheses.

*H3:* Opportunistic behavior attitudes of enterprise boundary spanners will positively and significantly influence their opportunistic behavior intentions.

*H4:* Opportunistic behavior subjective norms of enterprise boundary spanners will positively and significantly influence opportunistic behavior intentions.

TRA further explains that the intention is the direct antecedent of the behavior taken by the individual (Ajzen and Fishbein, 1977). Ng (2020) discovered that employees’ intentions to share knowledge have a positive effect on their behaviors. Furthermore, Siponen et al. (2014) proposed that employees’ intentions for information security behavior influence their actions positively. Furthermore, Mishra et al. (2014) demonstrated that green IT usage intentions influence green IT usage behavior. As a result, in collaborative innovation projects, boundary spanners’ opportunistic behavior intentions can influence opportunistic behaviors. Therefore, this study proposes the following hypothesis.

*H5:* Opportunistic behavior intentions of boundary spanners will positively and significantly influence opportunistic behaviors.

#### The Hypotheses of Mediating Effects

Statistically, consider the effect of the independent variable X on the dependent variable Y. If independent variable X affects Y through the variable M, then M is the mediating variable (Baron and Kenny, 1986). This study previously deduced that Guanxi will influence the opportunistic behavior attitudes of boundary spanners. Attitudes will also influence the opportunistic behavior intentions. As a result, it is possible to conclude that opportunistic behavior attitudes mediate Guanxi and opportunistic behavior intentions. Furthermore, this study hypothesized that Guanxi would influence the opportunistic behavior subjective norms of boundary spanners. Subjective norms of opportunistic behavior can also influence intentions to engage in opportunistic behavior. As a result, it can be assumed that subjective norms of opportunistic behaviors act as a mediator between Guanxi and opportunistic behavior intentions. As a result, this study proposes the following hypotheses.

*H6:* There is a mediating effect of opportunistic behavior attitudes of boundary spanners in the relationship between Guanxi and opportunistic behavior intentions.

*H7:* There is a mediating effect of the opportunistic behavior subjective norms of boundary spanners in the relationship between Guanxi and opportunistic behavior intentions.

**Figure 1:**
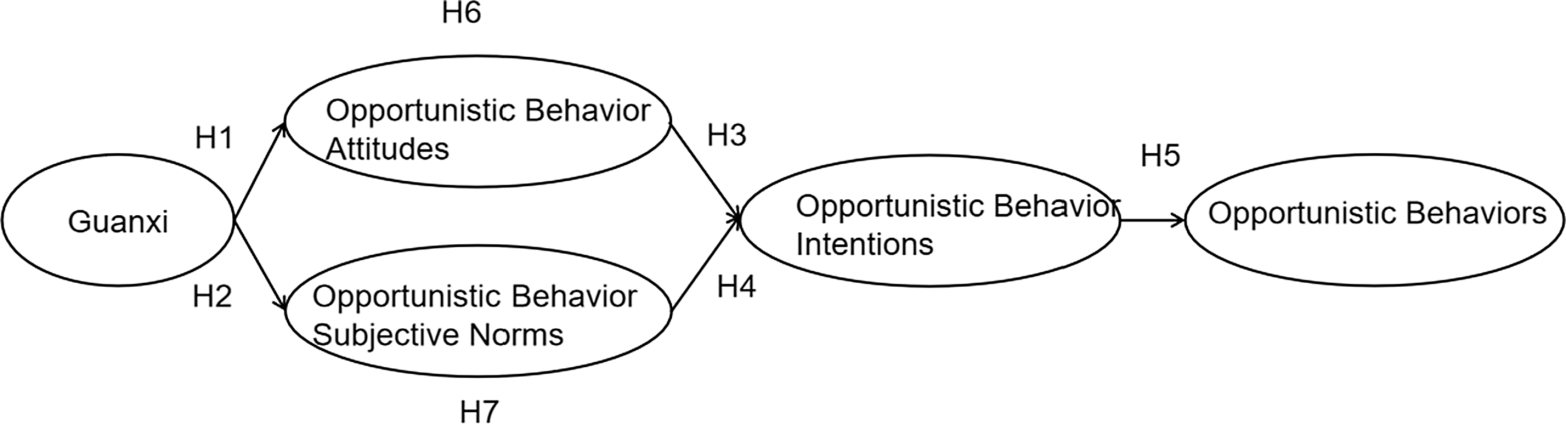
Theoretical framework of the research.

This study proposes the research model in Figure 1.

## Research Design

### Subjects and Sampling Method

This study was conducted in the vital automobile industry of Changchun City. There has been formed an automobile industry cluster around the vehicle manufacturer FAW. The automobile industry is a technology-intensive industry with a long industrial chain. As a result, collaborative innovation is critical for the growth of the automobile industry. The cluster is home to plethora of collaborative innovation projects. The respondents are the boundary spanners in the collaborative innovation projects acting as representatives and collaborating with employees sent by the partner enterprises. The research team is based in Changchun, which has strong Guanxi in the automobile industry. They can more easily reach and comprehend the boundary spanners. As a result, the intentional sampling method was used in the sampling design. After being introduced by social acquaintances, the researchers contacted corporate executives familiar with innovation in their companies. The researchers asked the executives to identify the boundary spanners in the collaborative innovation projects of their companies and describe their job responsibilities and main work content. On the basis of the descriptions, the researchers determined whether they were boundary spanners and asked boundary spanners to fill out questionnaires. The questionnaire survey had 1,102 respondents and ran from October 13 to November 21, 2021. After removing the questionnaires with excessive concentration, missing values, and concentration test questions, 542 questionnaires with a valid rate of 49.18% were obtained, providing a sufficient sample for statistical purposes.

### Description of Variables

The research variables in this study contain the essential personal background of boundary spanners, Guanxi, opportunistic behavior attitudes, opportunistic behavior subjective norms, opportunistic behavior intentions, and opportunistic behaviors. The operational definitions of research variables are as follows.

#### Boundary Spanner Personal Background

Basic personal data of boundary spanners were measured in the second part of the questionnaire, including five items, such as gender, education level, years of work experience, work position, and nature of the business property.

1. Gender: male, female.
2. Education level: junior high school and below, high school, college and university, and graduate school.
3. Years of work experience: <2, 3–5, 6–9, and > 10 years.
4. The nature of business property: state-owned enterprises, private enterprises, joint ventures, and foreign enterprises.
5. Work position: senior manager, middle manager, and general employee.

#### Questionnaire Design for the Measurement of Latent Variables

The researchers conducted a back-to-back selection of items based on the Chinese socio-cultural context, the contemporary Chinese way of being and thinking, and the scenario of collaborative innovation. Then, the research team had a face-to-face discussion about the selected items to identify alternative items. Following that, the study invited senior, middle, and junior staff members who had participated in corporate collaborative innovation projects. Experts and scholars in the fields of innovation and channel management were also invited to discuss and confirm the items chosen. The questionnaire was designed on a 7-point Likert scale, with “1” indicating strong disagreement and “7” indicating strong agreement. The questionnaire was gradually revised to perfection.

#### Measurement of Latent Variables

Regarding the measurement of Guanxi, this study compared the research of Lee and Dawes, (2005), Zhuang et al. (2010), Huang et al. (2011), and Zhang and Zhang (2013). All of the four studies are about the Guanxi of boundary spanners and are consistent with the present research context. Guanxi was measured using six items, derived from these four studies. Attitudes, subjective norms, and intentions toward opportunistic behaviors were adapted from Ng's (2020) research on employees’ knowledge-sharing behaviors. This study was improved to better fit the research topic of opportunistic behaviors. Luo (2006) provided the measurement of opportunistic behaviors and proposed two types of opportunistic behaviors: strong and weak. Boundary spanners are more likely to perform weak form opportunistic behaviors limited by work authority. This research measured six kinds of opportunistic behaviors adapted from the research of Luo (2006). The operational definitions and reference resource are shown in Table 1. The items of each construct are shown in Table 2.

**Table 1:** Operational definition and reference resources of each latent variable.

Variable	Operational definition	Reference
Guanxi	Boundary spanners from two parties often meet privately to have dinner or hang out, belong to the same circle, have close relationships just like relatives and friends, maintain a good relationship even if the collaborative innovation project is over, give “face” to each other and help each other based on personal relationships rather than work relationship.	Lee and Dawes, 2005; Zhuang et al., 2010; Huang et al., 2011; Zhang and Zhang, 2013
Opportunistic behavior attitudes	The perceptions of boundary spanners to adopt opportunistic behaviors in collaborative innovation projects, which is helpful to their work and company, will not harm the project and is valuable and wise for the company.	Ng, 2020
Opportunistic behavior subjective norms	The perception of boundary spanners’ supervisors, leaders, colleagues, family members, and friends on the opportunistic behaviors.	Ng, 2020
Opportunistic behavior intentions	The likelihood that boundary spanners would act opportunistically in the following collaborative innovation project undertaken, such as reluctant to share knowledge; concealing the key resources; misrepresenting their true level of competence; indifferent to the difficulties; will not give full effort; and will not meet the requirements of accuracy, completeness, and timeliness.	Luo, 2006; Ng, 2020
Opportunistic behaviors	The boundary spanners do not share their knowledge resources, hide the key resources needed by the partner, misrepresent the actual level of their competencies, are indifferent to the difficulties encountered by the partner, and do not give their best effort for the benefit of their company in the collaborative innovation projects.	Luo, 2006

**Table 2:** Measurement items of each construct.

***Guanxi (scaling from “Strongly disagree” to “Strongly agree” on a 7-point scale)***
GU1 The counterparts and I often meet privately to have dinner or hang out. GU2 I think the counterparts and I belong to the same circle. GU3 I have close relationships with my counterparts. They are just like my relatives and friends. GU4 Even if our collaborative innovation project is over, I will still maintain a good relationship with my counterparts. GU5 I give “face” to my counterparts, and they also give me face. GU6 The counterparts and I often help each other based on personal relationships rather than work relationships.
***Opportunistic behavior attitudes (scaling from “Strongly disagree” to “Strongly agree” on a 7-point scale)***
OBA1 I think it’s helpful to my work to adopt opportunistic behaviors. OBA2 I think it’s helpful to my company to adopt opportunistic behaviors. OBA3 I do not believe that adopting opportunistic behaviors will have a negative impact on collaborative innovation projects. OBA4 I think it’s valuable for my company to take opportunistic behaviors. OBA5 I think it’s wise for my company to adopt opportunistic behaviors.
***Opportunistic behavior subjective norms (scaling from “Strongly disagree” to “Strongly agree” on a 7-point scale)***
OBN1 My supervisors encourage everyone to act opportunistically for the interest of the company. OBN2 My leaders feel that I should act opportunistically for the interest of the company. OBN3 My colleagues think that I should act opportunistically for the interest of the company. OBN4 My family believe that I should act opportunistically for the interest of the company. OBN5 My friends think that I should act opportunistically for the interest of the company.
***Opportunistic behavior intentions (scaling from “Strongly disagree” to “Strongly agree” on a 7-point scale)***
OBI1 For the benefit of the company, I will be reluctant to share our knowledge with our partners in the upcoming collaborative innovation projects. OBI2 For the benefit of the company, I will conceal the key resources required by the partner in the next collaborative innovation projects that we undertake. OBI3 For the benefit of the company, I will misrepresent our true level of competence in the collaborative innovation projects that will be carried out. OBI4 For the benefit of the company, I will be indifferent to the difficulties encountered by my collaborators in the next collaborative innovation projects that we carry out. OBI5 For the benefit of the company, I will not give my full effort in the collaborative innovation projects to be carried out next. OBI6 For the benefit of the company, I will not meet the requirements of accuracy, completeness, and timeliness in disclosing information to collaborators in the next collaborative innovation projects that we undertake.
***Opportunistic behaviors (scaling from “Strongly disagree” to “Strongly agree” on a 7-point scale)***
OB1 I am sometimes reluctant to share our knowledge in collaborative innovation projects with other companies due to company interests. OB2 When collaborating with other companies on innovations, I sometimes hide key resources needed by the collaborators for the benefit of the company. OB3 For the benefit of the company, I sometimes misrepresent the actual level of my company competencies when working with other companies on innovations. OB4 For the benefit of the company, I am sometimes indifferent to the difficulties encountered by my collaborators. OB5 For the benefit of the company, I sometimes do not give my full effort in collaborative innovation projects. OB6 For the benefit of the company, I do not meet the requirements of accuracy, completeness, and timeliness in disclosing information to collaborators in the collaborative innovation projects.

## Results

### Descriptive Statistical Analysis

#### Frequency Distribution

The category elements of the 542 valid questionnaires include gender, education, working years, enterprise ownership property, and position. Of the respondents, 61.3% are men. The enterprises may believe that men are more suitable to have a contract with collaborators. In terms of education, 88.4% of the respondents graduated from university or college. The respondents’ working years are mainly 6–9 years, accounting for 60.3% of the total. It implies that boundary spanners are always employees who have extensive experience in collaboration work. Of the respondents, 63.5% work in the private sector. It is possible to conclude that private enterprises will focus more on collaboration. Private enterprises may be more open to inter-enterprise cooperation and innovation, as well as more flexible in their innovation development. Because they can receive government support, state-owned enterprises are more likely to innovate independently. Of the respondents, 50.4% hold middle-level positions. Middle-level employees may be better suited for the role of boundary spanners. The data are shown in Table 3.

**Table 3:** Frequency distribution.

Variable	Lable	Frequency	Percent
Gender	Male	332	61.3
	Female	210	38.7
Education	High school or below	4	0.7
	College/University	479	88.4
	Master or above	59	10.9
Working years	≤2 years	11	2.0
	3–5 years	160	29.5
	6–9 years	327	60.3
	≥ 10 years	44	8.1
Enterprise’s ownership property	State-owned enterprise	87	16.1
	Private enterprise	344	63.5
	Joint ventures and Foreign companies	100	18.5
	Else	11	2.0
Position	High-level	32	5.9
	Middle-level	273	50.4
	Basic-level	237	43.7
*N* = 542			

#### Item Statistical Analysis

The mean values and standard deviation values of items of each construct were shown in Table 4. The lowest average score of the item is 2.825. The item is “I have close relationships with my counterparts. They are just like my relatives and friends” measuring the Guanxi construct. The highest average score is 4.758. The item is “I think it’s helpful to my company to adopt opportunistic behavior,” which is used to assess opportunistic behavior attitudes.”The standard deviation with the lowest value is 1.348. The item is “I have close relationships with the counterparts. They are just like my relatives and friends” measuring Guanxi. The item with the lowest average and standard deviation is the same. The maximum standard deviation is 1.77. The item is “For the benefit of the company, I do not meet the requirements of accuracy, completeness, and timeliness in disclosing information to collaborators in the collaborative innovation projects” of opportunistic behaviors.

**Table 4:** Item statistical analysis and convergent validity.

Construct	Item	Item reliability		Construct reliability	Convergence validity
		Std.	SMC	CR	AVE
Opportunistic behavior	OB1	0.649	0.421	0.866	0.520
	OB2	0.695	0.483		
	OB3	0.813	0.661		
	OB4	0.679	0.461		
	OB5	0.723	0.523		
	OB6	0.754	0.569		
Opportunistic behavior attitudes	OBA1	0.820	0.672	0.896	0.634
	OBA2	0.842	0.709		
	OBA3	0.627	0.393		
	OBA4	0.852	0.726		
	OBA5	0.819	0.671		
Opportunistic behavior subjective norms	OBN1	0.670	0.449	0.880	0.597
	OBN2	0.693	0.480		
	OBN3	0.857	0.734		
	OBN4	0.841	0.707		
	OBN5	0.785	0.616		
Guanxi	GU1	0.646	0.417	0.869	0.528
	GU2	0.784	0.615		
	GU3	0.660	0.436		
	GU4	0.800	0.640		
	GU5	0.692	0.479		
	GU6	0.760	0.578		
Opportunistic behavior intentions	OBI1	0.714	0.510	0.912	0.634
	OBI2	0.782	0.612		
	OBI3	0.838	0.702		
	OBI4	0.806	0.650		
	OBI5	0.834	0.696		
	OBI6	0.795	0.632		

*Unstd., Unstandardized factor loadings; Std, Standardized factor loadings; SMC, Square multiple correlations; CR, Composite reliability; AVE, Average variance extracted*.

### Measurement Model Verification

#### Convergent Validity

This study followed the two-step approach of structural equation modeling (SEM) proposed by Anderson and Gerbing (1988) to estimate the measurement and structural model. The first step used confirmatory factor analysis to examine the construct reliability and validity of the measurement, and the second step checked the path effects and their significance of the structural model. Maximum likelihood estimation (MLE) was used to evaluate the measurement model in terms of factor loadings, measurement reliability, convergent validity, and discriminant validity.

Table 4 illustrates a summary of unstandardized factor loadings, standardized factor loadings, standard errors, significance tests, square multiple correlations, composite reliability, and average variance extracted (AVE). Fornell and Larcker (1981) proposed three indices for assessing convergent validity of measurement items: (a) item reliability of each measure or square multiple correlations, (b) composite reliability of each construct, and (c) the average variance extracted (AVE). The internal consistency of reliability of all indicators in a construct is referred to as composite reliability.

As Table 4 shows, all standardized factor loadings of questions are from 0.627 to 0.857 falling into a reasonable range. The results demonstrate all questions have convergent validity. All the composite reliability of the constructs ranging from 0.866 to 0.912, exceed 0.7 recommended by Nunnally and Bernstein (1994) indicating all constructs have internal consistency. Last, all AVE valued ranging from 0.520 to 0.634, exceed 0.5 suggested by Fornell and Larcker (1981) and Hair (1998) showing all constructs have adequate convergent validity.

#### Discriminant Validity

The research used average variance extracted (AVE) as the criteria for testing the discriminant validity of constructs. According to Fornell and Larcker (1981), if the square root of the AVE of a construct (the bold figures in Table 5) is larger than the Pearson correlative coefficients with other constructs (the figures under the diagonal), implying the construct is discriminant with other construct. The results in Table 5 show that most constructs are good discriminant validity.

**Table 5:** The result of discriminant validity analysis.

	Mean	OB	OBA	OBN	GU	OBI
OB	3.675	0.721				
OBA	4.471	0.388	0.796			
OBN	4.4526	0.425	0.755	0.773		
GU	3.521	−0.126	−0.160	−0.277	0.727	
OBI	3.264	0.727	0.405	0.436	−0.071	0.796

*The diagonal value is the square root of the average variance extracted of this latent variable; OB, opportunistic behaviors; OBA, opportunistic behavior attitudes; OBN, opportunistic behavior subjective norms; GU, Guanxi; OBI, opportunistic behavioral intentions*.

### Structural Equation Model

#### Model Fit

The research by Jackson et al. (2009) collected 194 SSCI papers and summarized 9 indicators to report model fit. Furthermore, when using SEM to analyze samples larger than 200, chi-square tends to be too large indicating poor fitness. As a result, the bootstrap method was used in this study to correct the model (Bollen and Stine, 1992). Table 6 displays the corrected model fit indexes’ results. All of the indexes are reasonable. The result indicates that the model fit is good.

**Table 6:** Model fit verification.

Fit indicators	Criteria	Fit indicators of research model
Chi-square		437.261
Degree of freedom		345
CFI	>0.9	0.990
RMSEA	<0.08	0.022
TLI	>0.9	0.989
GFI	>0.9	0.954
NFI	>0.9	0.989
χ^2^/*df*	<3	1.267
AGFI	>0.8	0.944

*CFI, Comparative fit index; RMSEA, Root mean square error of approximation; TLI, Tucker-lewis index (non-normed fit index); GFI, Comparative fit index; NFI, Normed fit index; χ^2^/df, Normed chi-square; AGFI, Adjusted goodness of fit index*.

#### Path Analysis

**Figure 2:**
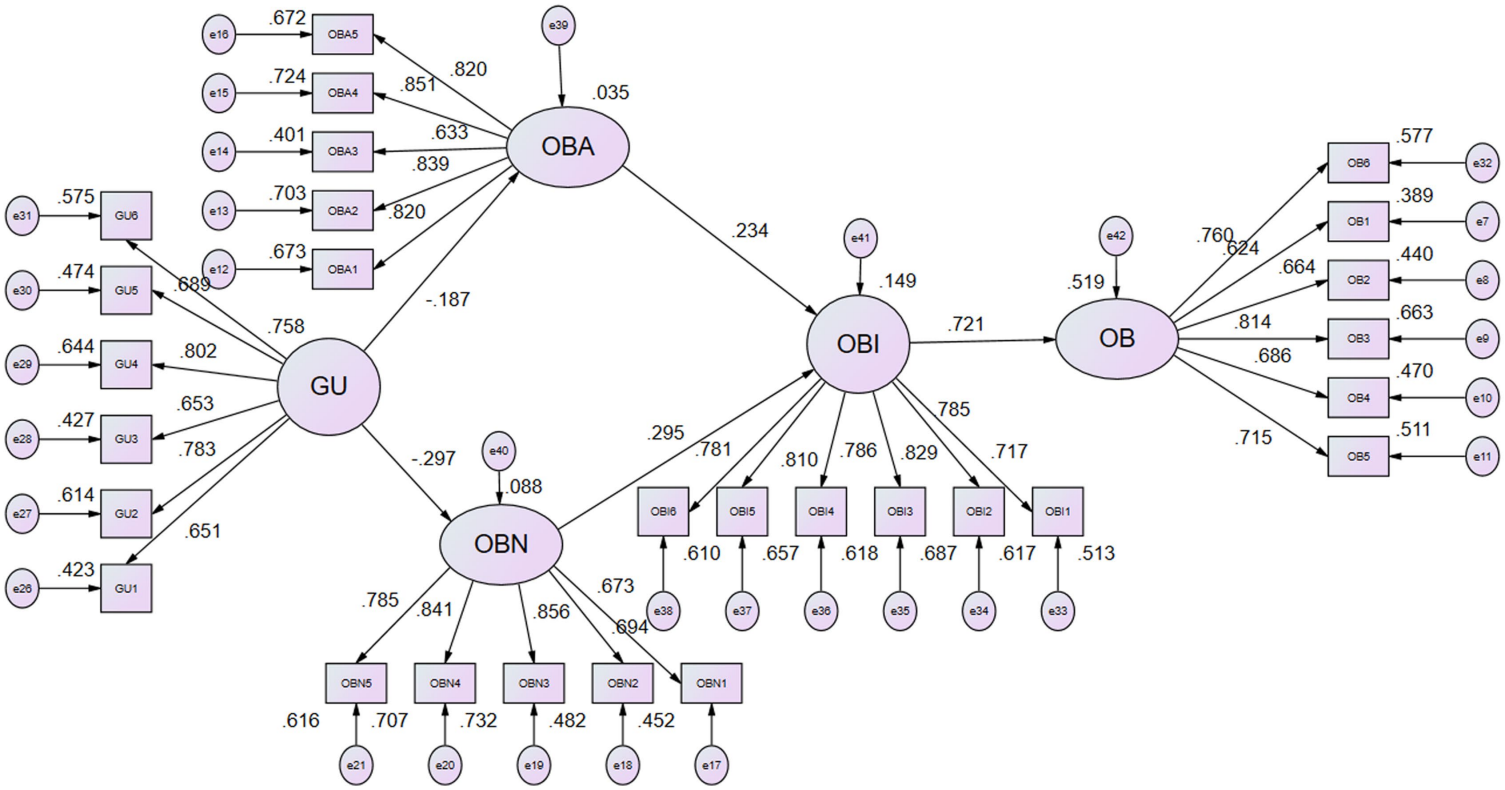
SEM statistic model.

Table 7 shows the path coefficient analysis for verification of the casual relationship between variables. The results show that Guanxi negatively influences attitudes and subjective norms toward opportunistic behaviors. The unstandardized regression coefficients from Guanxi to attitudes and subjective norms toward opportunistic behaviors are −0.212 and − 0.302, respectively. The values of *p* are <0.001, indicating that Guanxi has a significant influence on attitudes and subjective norms toward opportunistic behaviors. H1 and H2 are accepted. The findings also show that attitudes and subjective norms have a positive influence on intentions to engage in opportunistic behaviors. The unstandardized regression coefficients are 0.219 and 0.307. Both values of *p* are <0.001, indicating a significant impact. H3 and H4 are accepted. Intentions have a positive influence on behavior as well. The unstandardized regression coefficient is 0.639. The value of *p* is <0.001. It demonstrates that opportunistic behavior intentions have positively influence opportunistic behaviors significantly. H5 is supported. 3.5% of opportunistic behavior attitudes can be explained by Guanxi. 8.8% of opportunistic behavior subjective norms can be explained by Guanxi. Attitudes and subjective norms toward opportunistic behaviors can explain 14.9% of opportunistic behavior intentions. 51.9% opportunistic behaviors can be explained by intentions. Figure 2 shows the regression coefficients of the structural equation model. Figure 2 depicts the structural equation model’s regression coefficients.

**Table 7:** Regression coefficient.

DV	IV	Unstd.	S.E.	Unstd./S.E.	*p*-value	Std.	*R* ^2^
OBA	GU	−0.212	0.055	−3.829	0.000	−0.187	0.035
OBN	GU	−0.302	0.052	−5.784	0.000	−0.297	0.088
OBI	OBA	0.219	0.043	5.106	0.000	0.234	0.149
	OBN	0.307	0.050	6.088	0.000	0.295	
OB	OBI	0.639	0.055	11.555	0.000	0.721	0.519

*GU, Guanxi; OBA, Opportunistic behavior attitudes; OBN, Opportunistic behavior subjective norms; OBI, Opportunistic behavior intentions; OB, Opportunistic behaviors; DV, Dependent variable; IV, Independent variable; Unstd., Unstandardized regression coefficients; S.E., Standard error; Std., Standardized regression coefficients; R2, Explainable variations*.

#### Mediation Effect Analysis

Bootstrapping is a better method to test the mediation effect (Williams and MacKinnon, 2008). The research used bootstrapping as the repeated sampling method to test the indirect effects. Table 8 shows the results of Guanxi influencing behavior intentions through attitudes and subjective norms toward opportunistic behaviors. The first line of evidence shows Guanxi influencing behavior intentions *via* attitudes toward opportunistic behaviors. The confidence intervals range from −0.095 to −0.020, with a value of *p* is <0.001. Both confidence intervals are <0. The second line results show Guanxi influencing behavior intentions toward opportunistic behaviors *via* subjective norms. The confidence intervals range from −0.153 to −0.053, with a value of *p* <0.001. Both confidence intervals are<0, too. The two indirect effects are supported. H6 and H7 are supported.

**Table 8:** The analysis of indirect effects.

Effect	Point estimate	Product of coefficients			Bootstrap 1,000 times	
					Bias-corrected 95%	
		S.E.	*Z*-value	*p*-value	Lower bound	Upper bound
**Indirect effect**						
GU → OBA → OBI	−0.046	0.018	−2.556	0.001	−0.095	−0.020
GU → OBN → OBI	−0.093	0.024	−3.875	0.001	−0.153	−0.053

*GU, Guanxi; OBA, Opportunistic behavior attitudes; OBN, Opportunistic behavior subjective norms; OBI, Opportunistic behavior intentions; OB, Opportunistic behaviors*.

## Discussion

The research investigated the influence of Guanxi between boundary spanners on the opportunistic behaviors of boundary spanners. On the basic of TRA, the study constructed a model including Guanxi, opportunistic behavior attitudes, opportunistic behavior subjective norms, opportunistic behavior intentions, and opportunistic behaviors. The data were analyzed using SEM after questionnaires were collected from the boundary spanners in collaborative innovation projects in the automobile industry in Changchun. All of the hypotheses were found to be true. The findings indicate that Guanxi would restrain behavioral intentions toward opportunistic behaviors through attitudes and subjective norms. Behavioral intentions can also strongly predict behaviors.

### Theoretical Contribution

#### The Influence of Guanxi on the Attitudes and Subjective Norms Toward Opportunistic Behaviors

For the measurement of Guanxi, the item “I have close relationships with my counterparts. They are just like my relatives and friends” has the lowest mean and standard deviation. The low score may show that boundary spanners do not have a too-close relationship with each other. The low standard deviation could imply that boundary spanners have highly consistent opinions. Guanxi’s average score is 3.521. The low score suggests that developing good Guanxi is not popular among boundary spanners in Changchun’s automobile industry collaboration projects. To avoid revealing core technology and knowledge, enterprises may not encourage boundary spanners to develop Guanxi in collaboration projects.

The research results make clear that Guanxi has a significant negative influence on attitudes toward opportunistic behaviors. The result is consistent with that of the previous research, arguing that one person in a good Guanxi will not hurt the other person (Galvin et al., 2021). Although opportunistic behaviors may benefit their companies, the boundary spanners weigh the benefits and costs and concluded that maintaining good Guanxi is more valuable. Guanxi between boundary spanners is stronger in a collaborative innovation project; attitudes toward opportunistic behaviors are weaker. The results also show that Guanxi negatively influences the subjective norms toward opportunistic behaviors. It is consistent with the principle of Chinese socialization that individuals always consider the judgment from others before taking action (Lee and Lee, 2017). Of the standardized regression coefficients for the influence of Guanxi on attitudes and subjective norms toward opportunistic behaviors, are −0.187 and − 0.297, respectively in the path analysis. It demonstrates that Guanxi has a strong inhibiting effect on subjective norms toward opportunistic behavior. It can also be concluded that boundary spanners are more concerned with their social standing.

#### The Influence of Attitudes and Subjective Norms on the Intentions Toward Opportunistic Behaviors

The average score of attitudes and subjective norms toward opportunistic behaviors are 4.471 and 4.452, respectively. The two scores are pretty close, indicating that boundary spanners’ evaluation of opportunistic behaviors is almost the same as the perceived evaluation of opportunistic behaviors by those around them. “I do not believe that adopting opportunistic behaviors will have a negative impact on collaborative innovation projects,” the item measuring opportunistic behavior attitudes, has the lowest score and the highest standard deviation. It means that boundary spanners believe that opportunistic behaviors will harm the collaboration project. In contrast, boundary spanners have wildly divergent viewpoints. The item measuring opportunistic behavior subjective norms, “My family believes that I should act opportunistically in the interest of the company,” also has the lowest score and the highest standard deviation. It can be concluded that family may not encourage boundary spanners to behave opportunistically. Parents constantly remind their children to be honest is a social phenomenon. Surprisingly, the boundary spanners` perception of their families’ attitudes toward opportunistic behavior is highly variable.

The results show that the attitudes and subjective norms toward opportunistic behaviors positively predict behavior intentions. The results are the same as TRA introduced. It is also consistent with the aforementioned results (Alzahrani et al., 2017; Ng, 2020), in which behavior attitudes and subjective norms can predict intention. In the path analysis of the influence of opportunistic behavior attitudes and subjective norms on behavior intentions, the standardized regression coefficients are 0.234 and 0.295, respectively. This means that subjective norms have a greater influence on behavioral intentions toward opportunistic behaviors. It is possible to conclude that the critical people who surround boundary spanners influence their intentions to behave more opportunistically, which is consistent with common sense and experience.

#### The Influence of Behavior Intentions on the Opportunistic Behaviors

The average score of the opportunistic behavior intentions is 3.264. It indicates that boundary spanners may not have a solid intention to behave opportunistically in a collaborative innovation project in the automobile industry in Changchun. The item with the lowest mean score and standard deviation is “For the benefit of the company, I will be indifferent to the difficulties encountered by my collaborators in the next collaborative innovation projects that we carry out.” It implies that boundary spanners are not unconcerned about the difficulties that collaborators face. Furthermore, this viewpoint is very consistent. Simultaneously, the average score of opportunistic behaviors is 3.675. The item “For the benefit of the company, I sometimes indifferent to the difficulties encountered by my collaborators” has the lowest mean score and lowest standard deviation. This result is the same as that of the measurement of opportunistic behavior intentions. The standardized regression coefficient in the path analysis of the influence of opportunistic behavior intentions on behaviors is 0.721. It suggests that behavior intentions can strongly predict behavior. Behavioral intention can significantly predict behavior, which is consistent with the aforementioned scholars’ research findings (Ng, 2020).

### Practical Contribution

This study confirmed that Guanxi between boundary spanners decreases their opportunistic behaviors. This means that individual-level Guanxi can be used as a form of informal governance of inter-firm opportunism. It can assist the firm reducing the risk of being harmed by opportunism from collaborative innovation counterparts. Managers of collaborative innovation projects should encourage boundary spanners to develop Guanxi appropriately, which reduces counterparts’ boundary spanners’ opportunistic behaviors. Good Guanxi can also help projects move along more quickly. It is also useful to make full use of resources, save time and increase collaborative innovative project output. Individual-level good Guanxi may promote inter-firm Guanxi, which can develop inter-firm trust (Shen et al., 2020). Trust between firms can lead to long-term cooperation between the two partners (Lee and Dawes, 2005). It can be seen that a good Guanxi between boundary spanners can help to improve the continuity of collaborative innovation.

Boundary spanners exhibit opportunistic behaviors in collaborative innovation projects always for the sake of corporate self-interest, as a form of pro-organizational unethical behavior (Zhang et al., 2021a). They are oblivious to the fact that such actions will have a negative influence on their businesses in the long run because it will harm the development of collaboration projects and the company’s reputation. As a result, in collaborative innovation projects, companies should steer boundary spanners away from opportunistic behaviors. The results suggested that the negative effect of Guanxi is stronger on subjective norms than on attitudes. Furthermore, the subjective norms also have a more significant negative effect on opportunistic behavior intentions than on attitudes. It can also be confirmed that subjective norms have a greater influence than attitudes. When considering behaving opportunistically, boundary spanners consider the opinions of important people around them. If the enterprises do not want their boundary spanners to act opportunistically, they can take advantage of the influence of subjective norms on boundary spanners. Managers can emphasize that opportunism is not encouraged when working with other parties. Enterprise should also foster an atmosphere of integrity, which leads boundary spanners to believe that dishonest employees are not popular in the workplace.

### Limitation and Future Research

First, all the samples in this study were in the automobile industry in Changchun, Jilin Province, China. So the findings have limited applicability to the opportunistic behaviors of boundary spanners in other regions and industries. Future research could take a broader sample of regions and industries. It can, for example, contrast differences between different regions of China or between eastern and western societies. Second, this study confirmed the hypothesis that Guanxi has a negative influence on opportunistic behavior attitudes and subjective norms. However, the low *R*^2^ value indicates that the explanatory power is low.

For future research, scholars can try the following aspects. First, because China’s economic and social development varies widely across regions, there may be regional differences in the effects of Guanxi on opportunistic behaviors. Therefore future comparative studies should be conducted in different regions. Future research should be carried out in other industry and region. Second, future research should include more antecedent variables that may influence the attitudes and subjective norms of boundary spanners’ opportunistic behaviors to improve the model’s explanatory power. For example, their loyalty to the organization, position, and working ability may all have a significant influence on their opportunistic behaviors. Third, existing research has examined the firm-level governance forces on opportunistic behaviors, such as contractual governance and managerial governance to organize and restrict firm behavior (Luo et al., 2015). It will require further examination of the interaction between firm-level governance forces and individual-level Guanxi. Finally, boundary spanner behaviors are also influenced by the environment. As a result, future research should assess the effect of organizational-level factors, such as organizational culture and internal control mechanisms. Inter-organizational-level factors, such as inter-organizational dependencies, inter-organizational trust, and collaboration model, should be investigated as well.

## Data Availability Statement

The original contributions presented in the study are included in the article/supplementary material, further inquiries can be directed to the corresponding authors.

## Ethics Statement

Ethical review and approval were not required for the study on human participants in accordance with the local legislation and institutional requirements. Written informed consent was obtained from all subjects involved in the study.

## Author Contributions

S-kZ and J-mC: conceptualization, formal analysis, investigation, methodology, validation, and writing-original draft and writing, reviewing, and editing. All the authors have read and agreed to the published version of the manuscript.

## Funding

This study received funding from National Natural Science Foundation of China (NSFC) (Grant no. 72174073).

## Conflict of Interest

The authors declare that the research was conducted in the absence of any commercial or financial relationships that could be construed as a potential conflict of interest.

## Publisher’s Note

All claims expressed in this article are solely those of the authors and do not necessarily represent those of their affiliated organizations, or those of the publisher, the editors and the reviewers. Any product that may be evaluated in this article, or claim that may be made by its manufacturer, is not guaranteed or endorsed by the publisher.
